# Assessment of the Presence of Soil Lead Contamination Near a Former Lead Smelter in Mombasa, Kenya

**DOI:** 10.5696/2156-9614-9.21.190307

**Published:** 2019-03-14

**Authors:** Bret Ericson, Victor Odongo Otieno, Cecelia Nganga, Judith St. Fort, Mark Patrick Taylor

**Affiliations:** 1 Pure Earth, New York, NY, USA; 2 Department of Environmental Sciences, Faculty of Science and Engineering, Macquarie University, Sydney, Australia; 3 School of Environmental and Earth Sciences, Pwami University, Kilifi, Kenya

**Keywords:** lead exposure, LMICs, informal settlements

## Abstract

**Background.:**

The informal settlement of Owino Uhuru near an abandoned lead smelter attracted international attention due to an apparent lead poisoning event. Despite this attention, the environmental data collected to date do not indicate high levels of residual contamination.

**Objectives.:**

To further confirm previous findings and determine any necessary risk mitigation measures, an assessment of surface soil lead concentrations was conducted in the community.

**Methods.:**

Investigators carried out an assessment of the soil in a ~12,000 m^2^ section of the Owino Uhuru neighborhood over the course of a single day in June 2017 with the assistance of community leaders. Fifty-nine *in situ* soil measurements were taken using an Innov-X tube-based (40 kV) alpha X-ray fluorescence instrument (pXRF).

**Results.:**

The assessment found that mean surface soil lead concentrations in areas conducive to exposure were 110 mg/kg (95% CI: 54–168); below United States Environmental Protection Agency and the Environment Canada screening levels of 400 mg/kg and 140 mg/kg, respectively.

**Conclusions.:**

There is likely no current need for risk mitigation activities in the community. These results could inform discussions on the allocation of public health spending.

**Competing Interests.:**

The authors declare no competing financial interests. BE, VOO, CN and JSF are employees of Pure Earth. MPT sits on the Editorial Board of the Journal of Health and Pollution.

## Introduction

Lead smelting is a significant source of global soil contamination.^1–4^ Surface soil lead concentrations in residential areas can result in exposure to humans through pica (hand-to-mouth) behavior and the inhalation and ingestion of contaminated soil and dust.^5^,^6^ Accordingly, several studies have demonstrated that blood lead levels (BLLs) are strongly associated with soil lead concentrations.^6–11^ There is currently no known safe level of exposure to lead, although the United States Centers for Disease Control (CDC) utilizes a reference dose of 5 μg/dL.^12^,^13^ Childhood lead exposure can result in intelligence quotient decrement, decreased lifetime earnings and higher rates of aggravated assault, among other adverse outcomes.^14–18^ Early life exposures have also been shown to not remit with age.^17^,^19^ Although rare, extreme acute lead exposure can result in encephalopathy and death.^20^ Lead exposure in adults most significantly results in increased incidence of heart disease, even at low levels of exposure.^20^,^21^ The Institute for Health Metrics and Evaluation (IHME) estimates that lead exposure accounted for 540,000 deaths globally in 2016.^22^

Over the course of the 20th century, environmental lead contamination was most strongly associated with the use of tetraethyl lead additives in gasoline.^23–26^ In the United States, lead-based paint was also a significant source of exposure.^27^,^28^ Following the cessation of the use of lead in these two common products, blood levels have fallen significantly. For example, in the United States, average childhood blood lead declined from around 15 μg/dL in the mid-1970s, falling to < 1 μg/dL today.^27^,^29^ Elsewhere in high-income countries, persistently elevated environmental and biological lead levels continue to be documented around mining and smelting locations.^30^,^31^

In low- and middle-income countries (LMIC), current major sources of lead exposure include traditional ceramic glazes and the manufacture and recycling of lead-acid batteries, particularly when conducted in an informal setting.^32–35^ Discrete lead poisoning events have also been identified at mining and smelting locations.^2^,^36–38^

In Kenya, elevated environmental and blood lead concentrations have been documented in occupational settings.^39^ However, there is a paucity of information about exposures in the home or in residential areas. Data collected as part of the Pure Earth (New York, NY, USA) Toxic Sites Identification Program indicate that domestic lead exposure may be significant.^40^ The program has identified and conducted rapid assessments of 60 discrete lead contaminated sites located in residential areas in Kenya since 2009.

**Figure 1:**
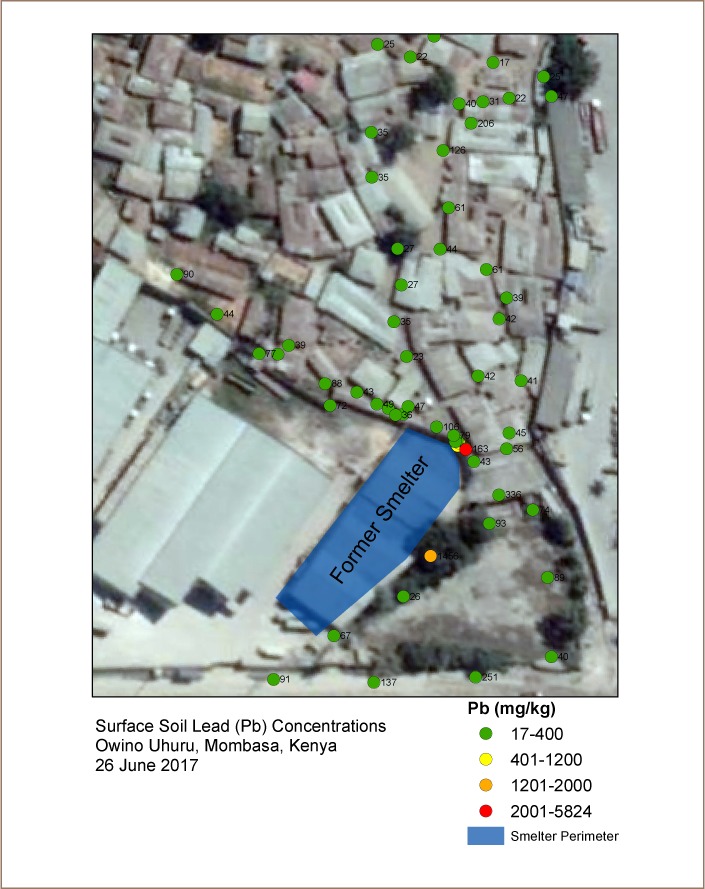
Location and concentration of in-situ X-ray fluorescence instrument surface soil lead measurements in Owino Uhuru

A lead poisoning event in 2014 centered around a lead smelter in the coastal city of Mombasa garnered international attention.^41–45^ News reports documented elevated BLLs and three deaths in the worker population at the refinery.^46^ Limited reports of elevated BLLs were also later found in Owino Uhuru, an informal settlement of approximately 3,000 residents with an area of 28 000 m^2^ bordering the northern wall of the facility (*Figure 1*). A 2010 study of three children found BLLs of 12, 17 and 23 μg/dL, while a later report of a separate child found a BLL of 32 μg/dL.^43^,^46^ A community leader in Owino Uhuru and former employee of the smelter was awarded the prestigious Goldman Environmental Prize for her work in raising public awareness that would eventually lead to the closure of the facility in 2014.^45^,^47^ The community subsequently filed a lawsuit against the national government for USD 1.5 million in compensation for its failure to monitor emissions from the smelter.^48^

Following the closure of the facility in 2015, a study of environmental lead concentrations and community BLLs was carried out jointly by the Kenya Ministry of Health and the CDC. The study found a geometric mean surface soil lead concentration in the community of 146.5 mg/kg (geometric standard deviation (GSD): 5.2) and dust lead loadings in homes of 1.5 μg/ft^2^ (GSD: 12.3).^49^ These results were significantly below applicable United States Environmental Protection Agency (USEPA) screening levels for residential soil and household dust of 400 mg/kg and 40 μg/ft^2^, respectively.^50^ In contrast with the low environmental levels, the study found a geometric mean BLL in children of 7.4 μg/dL (GSD: 1.9) in the community, exceeding both the CDC reference dose of 5 μg/dL and the BLLs found in a control neighborhood away from the facility of 3.7 μg/dL (GSD: 1.9).^13^,^49^

**Abbreviations**
*BLLs*: Blood lead levels
*CDC*: Centers for Disease Control and Prevention
*GSD*: Geometric standard deviation
*IEUBK*: Integrated Exposure Uptake Biokinetic model for children
*LMIC*: Low- and middle-income countries
*pXRF*: Alpha X-ray fluorescence instrument
*USEPA*: United States Environmental Protection Agency

Importantly, while the BLLs found in Owino Uhuru exceeded CDC guidelines, they were somewhat lower than those reported in communities near lead smelters elsewhere in LMICs. Researchers working near lead smelters in the Dominican Republic, Mexico, Senegal and Vietnam, for example, have reported population-wide BLLs in children of 71 μg/dL (arithmetic mean), 27.6 μg/dL (median), 40.4 μg/dL (median), and 129.5 μg/dL (mean), respectively.^2^,^51–54^

Despite the absence of evidence of residual contamination, the site continues to attract national and international attention.^55^,^56^ To further assess the veracity of the claims of contamination and provide the basis for necessary human health intervention strategies, an investigation of surface soil lead concentrations was carried out in Owino Uhuru in June 2017. This type of assessment forms the evidence-based rationale for any subsequent actions required to mitigate potential risk of harm arising from soil and dust contamination. Moreover, the results of the assessment in the context of its high profile potentially provide insight into the setting of public health priorities.

The results of this assessment are presented along with a simple air deposition model developed to estimate likely surface soil lead concentrations resulting from smelter emissions during its operation. The results of these models were used to estimate BLLs in children and adults in the absence of population data on exposures.

## Methods

Investigators carried out an assessment over the course of a single day in June 2017 with the assistance of community leaders. Fifty-nine *in situ* soil measurements were taken using an Innov-X tube-based (40 kV) alpha X-ray fluorescence instrument (pXRF) over a ~12 000 m^2^ section of the Owino Uhuru neighborhood that is adjacent to the facility. The pXRF has a lower detection limit of 5 mg/kg.^57^ Fifty-seven measurements were taken directly from surface soil, while two were taken at a depth of 10 cm. Two of the surface soil measurements were taken from an area within the perimeter wall of the smelter, which are unlikely to be accessed by humans and thus are not indicative of community exposure. The pXRF was calibrated before the assessment using an alloy-grade 316 steel clip and measurement accuracy was evaluated by assessment of a National Institute of Standards and Technology (NIST) standard (2702: Inorganics in Marine Sediment) during the assessment.^58^ The NIST reference material contains a known value for lead of 132.8 mg/kg. The pXRF measurement of this material found a value of 137 mg/kg (+/−10) for lead and was thus within acceptable range. The inside of the facility was not accessible and was not assessed.

### Spatial and statistical analysis

Latitude and longitude for each sample point were collected using World Geodetic System 1984 format using a Garmin eTrex 10 with an accuracy of < 3 meters.^59^ Spatial and statistical analyses were performed using ArcMap 10.5 and Stata 15.^60^,^61^ Basic descriptive statistics of the data were generated to assess exposure. In addition, simple linear regression was conducted to assess any relationship between lead concentration and proximity to the smelter.

### Aerial deposition model

To determine soil lead concentrations resulting from aerial emissions, a simple algorithm was developed based on known deposition rates of lead smelters in different settings. To determine the spatial extent of lead deposition, a Gaussian plume model was used to estimate the likely distributions in the Hybrid Single-Particle Lagrangian Integrated Trajectory (HYSPLIT) model developed by the United States Oceanic and Atmospheric Administration.^62^ Using contemporaneous meteorological data and the inputs set out below, the HYSPLIT model indicated that deposition was uniform within a range of 750 m of the smelter in all directions. The entire residential area of the Owino Uhuru falls within 300 m of the smelter stack. Thus, deposition was assumed to be uniform across the community.

A number of studies indicate that the accumulation of lead in soil is additive, meaning that concentrations increase proportionate to deposition.^63–65^ Alloway sets out a simple mass balance equation where background concentrations are increased by the accumulation of metals from various sources with any reductions occurring due to crop removal, leaching, volatilization or erosion. In the case of Owino Uhuru, as no agriculture is present in the assessed areas, crop removal would not be a relevant factor. Similarly, lead is highly immobile in soil and is not volatile (1.77 mm mercury at 1,000°C), thus leaching and volatilization would not appreciably affect the net concentration of lead in soil.^66^,^67^ Equation 1 sums the total estimated inputs and only accounts for limited migration.

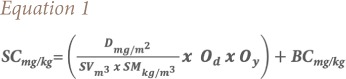
*where*

**SCmg/kg** equals surface soil lead concentration
**D_mg/m^2^_** equals deposition rate of lead in mg/m^2^ per day
**O_d_** equals number of days of operation per year
**O_y_** equals number of years of operation
**SV_m^3^_** equals volume of soil in m^3^
**SM_kg/m^3^_** equals mass in kilograms of one unit of soil
**BC_mg/kg_** equals background soil lead concentration

Deposition rate inputs were based on van Alphen's study of an area surrounding a lead smelter in Port Pirie, New South Wales, Australia. This study found a mean deposition rate of 18.8 mg/m^2^/day within 600 m of the smelter with maximum deposition rate of 299 mg/m^2^/day.^68^ These values are higher than those documented elsewhere. Studies at lead smelters in Arnhem (the Netherlands), El Paso (Texas, USA), Hoboken (Belgium), Missouri (USA), and Port Pirie (Australia) found deposition rates ranging from 0.1–17.5 mg/m^2^/day, for example.^30^,^68–72^ There are no known studies of deposition rates at rudimentary smelters like the one operated by the Mombasa facility, thus the selection of the highest deposition rates identified in the literature is intended to best approximate the poor conditions present at the facility. Similarly, a conservative stack height of 10 m was used for the purposes of modeling deposition.

Days of operation were assumed to be 260 days per year based on 5 days of operation per week for 52 weeks. The time period of the smelter operation was set at 10 years, based on news reports, which were the only available data.^46^

To determine the relevant mass of soil, a volume was calculated based on a likely penetration of deposited lead to a maximum depth of 2 cm. The 2 cm value is based on studies of the isotopic composition of soil lead at a smelter in Mount Isa, Australia.^73^,^74^ Mackay et al. found that lead found below this depth tended to be associated with naturally occurring deposits, rather than aerial deposition from the smelter.^73^ The soil type at the site is Haplic Lixisol with an approximate clay, silt and sand content of 18%, 27% and 55%, respectively and a mass of roughly 1.4 grams/cm^3^.^75^ These soils are conducive to metals mobility more generally, as discussed below. For the purpose of the sensitivity analysis the model was also run with a depth of 5 cm. Studies at a smelter in Boolaroo, Australia found deposited lead at a maximum depth of 5 cm with 80% less lead in the lower 2.5 cm than the top 2.5 cm.^76^

Background lead concentrations for the study area were not available. As an alternative, the mean background lead concentration for the earth's crust of 17 mg/kg was used.^77^ For context, crustal lead concentrations average 25.8 mg/kg in the United States and range from 8.4–40 mg/kg in Europe and the United Kingdom.^65^,^78^

### Blood lead level assessment

To estimate BLLs for children, the USEPA Integrated Exposure Uptake Biokinetic model for children (IEUBK) was used.^7^ Following IEUBK guidelines, default values for lead from all exposure pathways were used and measured *in situ* soil concentrations were entered.^79^ Default ingestion rates were then adjusted upward to account for higher ingestion rates in LMICs (250–400 mg/day).^10^,^80^ Results were also calculated using the default values (85–135 mg/day). For adults, the USEPA Adult Lead Methodology was used.^7^ Again, results were calculated using both default and augmented ingestion rates to account for increased exposure in LMICs (50–200 mg/day). Additionally, exposure duration was increased to account for a residential setting, as the Adult Lead Methodology's default values were intended for occupational exposures.

The IEUBK model was also used to estimate likely environmental Pb levels in air and soil required for a hypothetical 2-year-old child to have a BLL of 20 μg/dL. The IEUBK model assigns this age a higher BLL than younger or older age groups. It was selected to provide the most sensitivity to environmental levels.

## Results

The results of both the *in situ* surface soil measurements and aerial deposition modeling indicate that environmental lead levels in Owino Uhuru are within or slightly above US regulatory screening levels and generally consistent with urban areas globally.

### Surface soil assessment

The mean surface soil lead concentration in the areas assessed with the pXRF was 224 mg/kg (95% CI: 15–434). The median value was 47 mg/kg. Table 1 presents the summary results of the surface soil assessment. Four samples (7%) tested above 400 mg/kg, the USEPA screening level for bare soil where children play.^81^ Those four samples had the following concentrations: 582, 871, 1456 and 5824 mg/kg. The highest and third highest samples (5,824 and 871) were taken from an enclave in the perimeter wall of the smelter that is unlikely to be regularly accessed by humans and thus are not indicative of community exposure. Removing these from the likely exposure scenario results in a mean surface soil concentration of 110 mg/kg (95% CI: 54–168), below USEPA and Environment Canada screening levels of 400 mg/kg and 140 mg/kg, respectively.^81^,^82^ Kenya has not yet developed its own guidance values for soil metal concentrations, including lead. Two samples were collected at a depth of 10 cm adjacent to the highest reading (5,824 mg/kg) for the purpose of assessing possible migration. These readings were 43 and 72 mg/kg, indicating insignificant down profile migration of lead from surface soils, which is consistent with studies of atmospherically deposited smelter soil lead contamination elsewhere.^73^,^74^

**Table 1:** Descriptive Statistics of Surface Soil Concentrations in Owino Uhuru

No. of measurements	59		
	Mean (SD)	Median (IQR)	Range
Concentration (mg/kg)	219 (776)	47 (36,91)	17–5824

Abbreviations: SD, Standard deviation; IQR, Interquartile range.

Within the targeted 12,000 m^2^ sample area, soil lead measurements were spaced an average of 9.4 m apart (95% CI: 7.8–11.2) (*Figure 1*). There was no statistically significant association between proximity to the smelter and soil lead concentrations (p<0.05). The mean soil lead concentration of the eight samples taken within 3 m of the facility perimeter wall was 1,026 mg/kg (95% CI: 611–2663). The mean for the eight samples taken from 3 m to 10 m was 231 mg/kg (95% CI: 183–646) and the mean for the 43 samples taken beyond 10 m was 66 mg/kg (95% CI: 47–86). The sample taken closest to the smelter site was at the base of the perimeter wall, while the furthest was taken at a distance of 130 meters.

### Aerial deposition model results

Using van Alphen's mean deposition rate of 18 mg/m^2^/day and a 2 cm estimate for the likely maximum penetration of lead into surface soil resulted in an additional accumulation of 0.64 mg/kg/day within 750 meters of the facility while it was operating. Using the same deposition rate and the less conservative surface soil penetration estimate of 5 cm results in an additional accumulation of 0.26 mg/kg/day. These rates would have resulted in a surface soil concentration of 686–1,688 mg/kg after ten years of operation. To arrive at the mean value identified in pXRF sampling of 110 mg/kg, a daily deposition rate of 1–2.51 mg/m^2^/day (0.036 mg/kg/day) would be required.

### Blood lead level assessment

Current BLLs for 0- to 7-year-olds were estimated to be from 1.4–2.4 μg/dL using the default ingestion values in the IEUBK and 2.7–5.1 μg/dL using the augmented values. Current BLLs of adults were estimated to be 1.8–2.6 μg/dL, depending on the ingestion rate used. For surface soil exposure to result in a BLL of 20 μg/dL in 2-year-olds, an approximate surface soil concentration of ~2,500 mg/kg would be required with default ingestion values and ~850 mg/kg with the augmented values. With regard to air concentrations, a level of ~24 μg/m^3^ would be required for a 2-year-old to have a BLL of 20 μg/dL, assuming a soil lead concentration of 110 mg/kg.

## Discussion

Soil lead levels in Uhuru Owino seem to fall within internationally accepted screening levels and are at or below mean values in other cities globally. Abuja (Nigeria), Boston (USA), Brisbane (Australia), Glasgow (UK) and Stockholm (Sweden), for instance, have all been reported as having average city-wide soil concentrations exceeding 200 mg/kg.^83^ In Owino Uhuru, the average soil lead concentration in accessible areas was 110 mg/kg.

The surface soil lead levels currently present in Owino Uhuru are unlikely to produce elevated BLLs, although elevated BLLs were reported at the site during operation or shortly after.^7^,^43^,^49^ One possible explanation for the discrepancy could be a decline in surface level lead concentrations over time due to migration, meaning that current surface soil lead concentrations are not representative of soil lead concentrations while the smelter was operating. Lead is generally immobile in most soils, taking perhaps 700 years to halve in concentration in certain soil types.^66^ A number of factors can influence its mobility, including pH, cation exchange capacity and texture.^84^ Low cation exchange capacity (<2 cmol(+) kg), low pH, and a sandy texture are all associated with increasing lead mobility.^85^,^86^

However, even in locations where all or some of these conditions are present, very limited mobility of lead through soil profiles has been reported.^87–89^ Teutsch et al., for example, found similar lead contamination profiles at the same location 15 years apart, with measurable but very small amounts of lead migrating at a rate of up to 1 cm/year.^89^ The Haplic Lixisol soil in Owino Uhuru has an approximate cation exchange capacity of 1.8 cmol(+) kg and a pH of 5.9.^75^ Its clay, silt and sand content are roughly 18%, 27% and 55%, respectively.^75^ Thus, while these soils are more amenable to migration than others, migration is unlikely to account for any significant difference in surface soil concentration over the 10-year period between the opening of the smelter and the execution of this study.

A second possible explanation for the discrepancy between past BLLs and the current environmental levels present is that the primary exposure pathway to the community while the smelter was operating was the inhalation of airborne lead. Air lead concentrations would have declined immediately following the closing of the smelter. Reducing airborne sources of lead exposure near smelters has been strongly associated with declines in BLLs.^76^,^90^,^91^ No data are available on airborne concentrations at the site, either currently or while the smelter was operating. Given the limited data on the smelter operations, it was beyond the scope the current study to model those concentrations. Elsewhere, air lead levels exceeding 1 μg/m^3^ have been associated with BLL measurements above 10 μg/dL in children.^92^ In Port Pirie, air lead concentrations were recorded at levels up to 21.44 μg/m^3^.^93^

A separate likely source of exposure is associated with the workplace. Several residents with elevated BLLs were reported to have worked at the smelter, and anecdotal evidence indicates that children spent time in the facility during work hours. Additionally, take-home risk, or workers' inadvertent transporting of material on their person from the workplace to the home environment, could have also played a significant role as has been documented in multiple settings.^94–96^ Interior surfaces were not assessed as part of this study, although those assessed as part of the Ministry of Health/CDC effort shortly after the closing of the facility found very low lead dust loadings.^49^

Given that the key sources of exposure were most likely associated with the operation of the facility, the need for mitigation work in Owino Uhuru may not be as pressing as presented elsewhere. The former facility very probably contains high levels of lead on site, which should be appropriately considered in any future land use plans.

### Informal housing

There is a significant shortage of housing in urban areas in Kenya, with approximately 56% of urban dwellers living in informal settlements.^97^ These settlements are often located on marginal lands, including areas prone to flooding or landslides.^97^ In the case of Owino Uhuru, the settlement is an area that is characterized by industrial uses.^98^ It is bordered on two of three sides by industrial facilities and is accessed primarily through industrial land. A master plan for the city currently under development envisions the reclamation of Owino Uhuru for industrial ends.^98^ Industrial activity can play a critical role in economic development in LMICs, while siting residential areas sufficiently distanced from heavy industry, including lead smelting, could mitigate the most significant exposures.^34^,^99^ Here, inhibiting the likely illegal occupation of industrial land may have mitigated much of the adverse impacts.

### Setting public health priorities

A number of factors potentially influence the allocation of public health resources. These could include political forces, societal values and economic justifications, among others.^100–102^ In response to the various challenges implicit in allocating resources in public health, there has been a general coalescence in recent decades around rational and transparent approaches. Foremost among these is the notion of evidence-based decision making, commonly articulated in burden of disease, cost effectiveness, or equity analysis approaches.^102^ These approaches seek to make the best use of finite resources through “the conscientious, explicit, judicious and reasonable use of modern, best evidence in making decisions.”^103^,^104^

Within global public health, it is arguable that the issue of environmental lead poisoning receives proportionately less attention relative to its impact than other public health risks. IHME, for instance, calculated that lead exposure attributed to 13.9 million disability-adjusted life years and 540,000 deaths globally 2016.^22^ As context, this amounts to about 0.6 % of all disability-adjusted life years and about 1% of all deaths globally in the same year.^22^ In addition, there are indications that this value may be an underestimation.^33^ Major sources of environmental lead poisoning globally include industrial mining and smelting operations, lead-based ceramic glazes, and the recycling of used lead acid batteries.^105^ Lead exposure also results in a range of adverse societal impacts, including increased rates of violence and decreased economic output, that are not captured in disease burden approaches.^15^,^106^,^107^ Despite this considerable impact, there is currently no international convention or multi-lateral funding mechanism to support work related to lead contamination, resulting in a resource-poor environment for potential implementers.

In the context of disproportionately limited funding for lead interventions, the importance of evidence-based decision making is augmented. Put differently, the burden of proof to justify interventions should be high. The residents of Owino Uhuru are subject to myriad environmental health and livelihood risks common to life in informal settlements.^108^ However, this study has found that those related to lead exposure were likely mitigated with the closing of the facility. The lead poisoning event in Owino Uhuru continues to receive national and international media attention despite the absence of current information regarding the health impact.^56^,^109^,^110^ In evaluating the relative importance of an intervention in Owino Uhuru versus similar projects elsewhere, policy makers should be encouraged to utilize evidence like that presented here to form the basis of their decisions.

### Limitations

A key limitation in the present study is the heavy reliance on pXRF measurements. The instrument was calibrated, and accuracy was confirmed in accordance with the manufacturer's instructions. When conducted in sufficient quantity, pXRF measurements have been shown to closely approximate wet chemistry techniques.^111^ However, no samples from this study were sent for laboratory analysis. Measurements of the NIST reference material during the assessment found that the pXRF was functioning within acceptable standards.

A similar limitation relates to the spatial distribution of the surface soil measurements taken in the present study. The assessment, which focused on the residential area of Owino Uhuru, did not determine concentrations in the industrial areas to the west, south, and east of the facility. Moreover, within Owino Uhuru, significant assessment gaps are evident in Figure 1, particularly to the northwest of the facility. While this area is unlikely to present elevated concentrations resulting from aerial deposition, it is possible that the area could be contaminated by other means such as manual deposition of waste. However, interviews with community members did not indicate that this was the case. Nevertheless, that this area was not assessed here represents a limitation.

A separate limitation of the study is its reliance on the IEUBK model to estimate BLLs. A previous study carried out at the site found comparable environmental levels, as well as a statistically significant increase in BLLs associated with residence in Owino Uhuru compared to a control neighborhood.^49^ Likewise, studies carried out elsewhere have found elevated BLLs resulting from similar environmental exposures.^112^

## Conclusions

Surface soil lead concentrations within Owino Uhuru were within international screening levels at the time of the study, and lower than many urban areas globally. As noted above, a soil concentration of 850–2,500 mg/kg would be required for a 2-year-old child to have a BLL of 20 μg/dL. It is unlikely that this concentration was ever present in Owino Uhuru. Accordingly, there did not appear to be a compelling need for off-site exposure mitigation work at the time of the assessment. Proper occupational and engineering controls and better siting of the residential area would likely have mitigated the lead poisoning event. Future use of the land should consider probable high contamination levels onsite. The results of this study could be used to inform the discussion on public health spending and to inform any future intervention at the site.
